# Assisted reproduction mediated resurrection of a feline model for Chediak-Higashi syndrome caused by a large duplication in *LYST*

**DOI:** 10.1038/s41598-019-56896-9

**Published:** 2020-01-09

**Authors:** R. M. Buckley, R. A. Grahn, B. Gandolfi, J. R. Herrick, M. D. Kittleson, H. L. Bateman, J. Newsom, W. F. Swanson, D. J. Prieur, L. A. Lyons

**Affiliations:** 1 Department of Veterinary Medicine and Surgery, College of Veterinary Medicine, University of Missouri, Columbia, MO 65211 USA; 2 Department of Population Health and Reproduction, School of Veterinary Medicine, University of California - Davis, Davis, CA USA; 3 Veterinary Genetics Laboratory, University of California – Davis, School of Veterinary Medicine, Davis, CA 95616 USA; 4 Omaha’s Henry Doorly Zoo and Aquarium, Omaha, Nebraska 68107 USA; 5 Center for Conservation and Research of Endangered Wildlife, Cincinnati Zoo and Botanical Garden, Cincinnati, Ohio, 45220 USA; 6 Department of Medicine and Epidemiology, School of Veterinary Medicine, University of California - Davis, Davis, CA 95616 USA; 7 Department of Veterinary Microbiology and Pathology, College of Veterinary Medicine, Washington State University, Pullman, WA 99164-7040 USA

**Keywords:** Animal breeding, Medical genomics, Gene duplication

## Abstract

Chediak-Higashi Syndrome (CHS) is a well-characterized, autosomal recessively inherited lysosomal disease caused by mutations in *lysosomal trafficking regulator* (*LYST*). The feline model for CHS was originally maintained for ~20 years. However, the colonies were disbanded and the CHS cat model was lost to the research community before the causative mutation was identified. To resurrect the cat model, semen was collected and cryopreserved from a lone, fertile,  CHS carrier male. Using cryopreserved semen, laparoscopic oviductal artificial insemination was performed on three queens, two queens produced 11 viable kittens. To identify the causative mutation, a fibroblast cell line, derived from an affected cat from the original colony, was whole genome sequenced. Visual inspection of the sequence data identified a candidate causal variant as a ~20 kb tandem duplication within *LYST*, spanning exons 30 through to 38 (NM_001290242.1:c.8347-2422_9548 + 1749dup). PCR genotyping of the produced offspring demonstrated three individuals inherited the mutant allele from the CHS carrier male. This study demonstrated the successful use of cryopreservation and assisted reproduction to maintain and resurrect biomedical models and has defined the variant causing Chediak-Higashi syndrome in the domestic cat.

## Introduction

Chediak-Higashi syndrome (CHS) (OMIM Accession: 214500) is a rare autosomal recessive disorder characterized in humans by severe immune deficiency, oculocutaneous albinism, bleeding tendencies, recurrent pyogenic infections, progressive neurologic defects and a lymphoproliferative syndrome. The most common cause of death from CHS is from recurrent infections or the development of an accelerated phase with hemophagocytic lymphohistiocytosis. Approximately 90% of deaths occur in the first decade of life, and those who survive into adulthood develop progressive neurological symptoms^1^. The disease was first described in the 1940s to early 1950s^2–6^ and has been characterized in a host of diverse species, including cow^7–10^, mink^10–14^, killer whale^15,16^, fox^17,18^ and domestic cat^19–23^ (OMIA: 000185-9913, 9733, 494514, 452646, 9685). Continuing studies of CHS models have demonstrated their value in deciphering delta storage pool deficiencies^24^, heritable platelet disorders^25,26^ and cellular cytotoxicity^27^. Bone marrow transplantation has been a viable option for management of human CHS for over 30 years^28–31^.

The genetic cause for CHS was first defined in rodents^32–36^, with the locus historically known as *beige* due to the associated hypopigmentation phenotype^33^. The human homolog of the mouse *beige* locus revealed the first causative mutations for CHS in humans^37,38^ and was defined as *lysosomal trafficking regulator* (*LYST)*^37^. In humans, *LYST* encodes a 3,801 amino acid protein (11.4 kb transcript), which regulates intracellular protein trafficking to and from the lysosome (GCID:GC01M235824). In many species with CHS, mutations have been consistently identified within *LYST*. These species include, human^39–42^, cow^43^, mouse^44,45^, rat^46,47^ and Aleutian mink^48^. Overall, in the public archive of human genetic variation and phenotypes (clinVar)^49^, over 40 different pathogenic variants have been identified in *LYST* in humans, including nonsense and missense mutations, as well as insertions and deletions.

**Figure 1:**
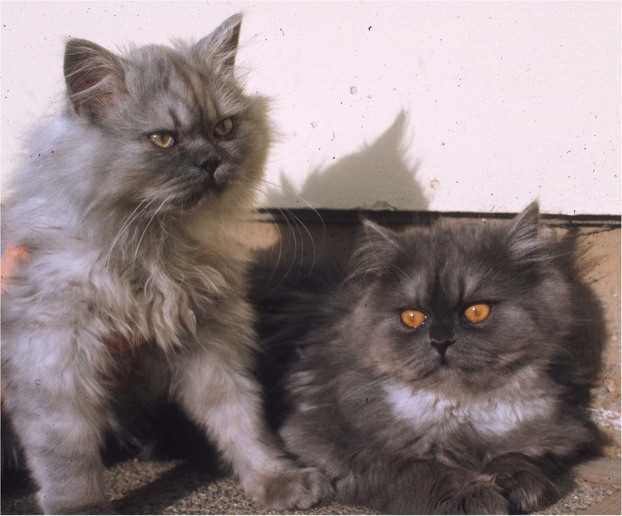
Chediak-Higashi syndrome in cats at 5.5 months of age. A symptom of Chediak-Higashi syndrome, the affected cat (left) has a much lighter coat color (hypopigmentation) than its non-affected littermate (right). Although both cats are genetically *aaB-C-D-E-I-ll*, the genotype for a solid black smoke longhaired cat. The affected cat is also photophobic and has pale yellow-green irises, instead of the normal copper color irises of its littermate.

Feline CHS was first noted in a lineage of Persian cats, and as in mice, the disease showed recessive autosomal inheritance and the sentinel presentation was hypopigmentation^19^ (Fig. 1). The clinical characterization of the feline model for CHS examined neutrophil and platelet functions and auditory and ocular pigmentation abnormalities. Genetic complementation analysis after interspecific somatic cell (fibroblast) hybridization between human and domestic cat cell lines demonstrated a lack of paracrystal formation, indicating homology of the diseases due to similar genetic defects^50^. However, the exact causative mutation for CHS in the cat model was never determined.

Long-term maintenance of cat heredity disease models, such as CHS, typically requires cats to be managed perpetually as living populations within university research colonies. Since many cat models originate from sporadic, *de novo* genetic mutations that were identified opportunistically as veterinary clinical cases, the demise of a specific model line may equate with its permanent extinction. However, semen and embryo cryopreservation, combined with artificial insemination or embryo transfer, can be used to preserve cat disease models, as commonly occurs with mouse research models^51,52^. For the CHS cat model, after nearly 20 years of research, the colonies at Colorado State and Washington State University could no longer be maintained and were lost to the research community. Fortunately, during the dissolution of the former CHS colony, an intact male (Smokey) was donated to the University of California, Davis. Smokey, a 16-year-old carrier for CHS, represented the only viable representative of the cat biomedical model for CHS. Therefore, the feline model for CHS provided an opportunity to apply newly advanced assisted reproductive techniques to resurrect a previously extinct feline disease model.

Here, semen from the viable CHS carrier of the original CHS colony, was successfully cryopreserved and used for artificial insemination (AI) to produce potential CHS carrier offspring. In addition, whole genome sequencing of fibroblast cell-lines derived from the original CHS cat colony helped identify a candidate causative mutation, a 20 kb tandem segmental duplication within *LYST* that spanned multiple exons. Finally, viable AI offspring were screened for the causative variant, demonstrating successful resurrection of a previously extinct feline model of a human disease and stability of the *LYST* mutation.

## Materials and Methods

### Cat management and sampling

All animal methods were carried out in accordance with PHS Policy on Humane Care and Use of Laboratory Animals, Animal Welfare Act Regulations, and Guide for the Care and Use of Laboratory Animals. Cat housing and husbandry, blood sample collection, assisted reproduction techniques and all other experimental protocols were approved under IACUC research protocols at the following institutions; the University of California- Davis (Protocol no. 16691), University of Missouri (MU) (Protocol no. 8787), and the Cincinnati Zoo & Botanical Garden (Protocol no. 05–064 and 11–102). Primary culture fibroblast cell lines of known carrier and affected cats from the CHS colony were thawed from LN_2_ storage. Using buccal swabs, DNA was collected from the male sperm donor, his viable offspring, and a random bred negative control. DNA was isolated by standard phenol - chloroform extractions methods^53^ or using DNeasy blood and tissue kit (Qiagen, Hilden, Germany)

### Semen collection and cryopreservation

For semen collection, the CHS-carrier male domestic cat was anesthetized with a combination injection (i.m.) of ketamine (5 mg/kg) and medetomidine (0.04 mg/kg), with anesthetic reversal at the conclusion of the procedure using atipamezole (0.08 mg/kg; i.m.). A rectal probe (1.0 cm diameter, 3 longitudinal electrodes) attached to an electrostimulator (PTE Electronics, Boring, Oregon) was used to deliver three controlled sets of electrical stimuli (2–5 V range; 20 to 30 stimuli/set) as previously described^54^. Recovered semen from each set was assessed for volume, pH, and presence or absence of spermatozoa. Raw spermic samples were assessed for motility (percent progressively motile; rate of progressive motility (RPM) on scale of 1 to 5) and an aliquot (5 µl) was fixed for later morphological assessment. The remaining semen was diluted 1:1 in culture medium (Feline Optimized Culture Medium with Hepes [FOCMH]^55^) and maintained in microcentrifuge tubes in the dark at room temperature until completion of semen collection. Spermic samples from each set were combined and then centrifuged (300 g, 10 min). The resulting sperm pellet was resuspended in Test-Egg Yolk (TEY; Irvine Scientific, Santa Ana, CA) containing 0% glycerol to a concentration of 50 × 10^6^ motile sperm/ml, suspended in a foam float in a 300 ml glass container (containing room temperature water), and placed into a refrigerator for 3.5 hours to cool to 5 °C. Cooled spermatozoa were diluted in three steps at 10 minute intervals with an equal volume of TEY (w/ 8% glycerol) to 25 × 10^6^ motile sperm/ml, and aliquots (30 µl each) frozen by pelleting into indentations in dry ice^56^. Frozen sperm pellets were plunged into liquid nitrogen and transferred into labeled cryovials for ground shipment to the Cincinnati Zoo’s Center for Conservation and Research of Endangered Wildlife (CREW) for storage within liquid nitrogen tanks.

### Ovarian synchronization and laparoscopic oviductal artificial insemination (LO-AI)

For ovarian synchronization and LO-AI, three female domestic cats (2–3 years of age) received oral progestin (altrenogest, Regumate^®^, Intervet/Merck Animal Health, Millsboro DE USA) daily mixed with moist cat food at a standard dosage (0.088 mg/kg body weight) for 39 consecutive days^57^. After four days of progestin withdrawal, females were treated with a combination regimen of equine chorionic gonadotropin (eCG; 100 IU; i.m.) followed 85 hours later with porcine luteinizing hormone (pLH; 1000 IU; i.m.), as previously described^58^. At 30–33 hours post-pLH, females were anesthetized and evaluated laparoscopically for the presence and number of mature follicles (>2 mm diameter) and corpora lutea. If mature pre-ovulatory follicles and/or freshly-formed corpora lutea were observed, frozen semen from the CHS-carrier male was thawed by depositing individual sperm pellets (8 pellets/LO-AI) into glass test tubes (1 pellet/tube) containing 100 µl of FOCMH for 30 sec in a 37 °C water bath. The thawed samples were combined and evaluated for sperm motility and concentration, as described above. One aliquot of a frozen-thawed sample was spread onto a microscope slide and later stained with FITC-PNA to assess acrosome status^59^. The combined sample then was centrifuged (600 g, 8 min), the supernatant removed and the concentrated sperm pellet resuspended in residual FOCMH to a small volume (18–20 µl) for insemination.

For LO-AI, an accessory trocar-cannula was placed into the right abdominal wall and grasping forceps used to secure and evert the ovarian bursa of the left ovary to visualize the oviductal ostium^58^. A polypropylene IV catheter (18 gauge; 32 mm in length) was inserted through the left abdominal wall perpendicular to the oviductal ostium and a blunted needle (22 gauge, 68 mm in length), containing 9–10 µl of concentrated sperm at its tip, was inserted through the catheter and directed through the oviductal ostium into the lumen of the oviductal ampulla. The sperm sample (9–10 µl) was deposited with slight air pressure delivered by an attached syringe as the insemination needle was slowly withdrawn from the oviduct. The LO-AI procedure was repeated with the right oviduct using a similar approach.

For pregnancy diagnosis, the females were assessed for fetal presence, number and viability via transabdominal ultrasound at 23 days post-AI, and pregnant females were reassessed at 41 and 59 days post AI to document fetal growth and viability. Pregnant females were allowed to carry offspring to term and monitored during natural parturition. If dystocia occurred during parturition, the pregnant queen was immediately anesthetized and the offspring were delivered by C-section.

### Whole genome sequencing

Cell line DNA from an affected cat (~3 ug) was submitted for whole‐genome sequencing to the McDonnell Genome Institute at Washington University. A TruSeq PCR‐free library (Illumina, San Diego, CA) was constructed with 450 bp insert size. The library was indexed with nine libraries from other cat samples from the 99 Lives project (http://felinegenetics.missouri.edu/99lives)^60^ pooled together in equal molar ratios based on their concentrations determined by qPCR. The samples were applied to a HiSeq X flow cell to generate ~1 Tb of data as 2 × 150 bp paired‐end reads. Reads were mapped to Felis_catus_9.0 using Burrows-Wheeler Aligner (BWA-MEM) tool^61^. *LYST* gene coordinates in Felis_catus_9.0 (https://www.ncbi.nlm.nih.gov/assembly/GCF_000181335.3/) were obtained from NCBI annotation release 104. The sequence reads aligned to the *LYST* gene were viewed using the integrative genomics viewer (IGV)^62^ and samplot (https://github.com/ryanlayer/samplot).

### PCR validation and Sanger sequencing

Primers were designed using the reference sequence surrounding the 5′, internal, and 3′ breakpoints of the duplicated region (Supplementary Table S1) to validate the segmental duplication in the cell lines and cats produced by AI. The reference sequences surrounding the left and right breakpoints of the duplicated region and the SINEC_Fc reference sequence, which was obtained from Repbase^63^, were used for primer design for breakpoint validation. Primers Right_BP_F and Left_BP_R spanned the central breakpoint present in the mutant allele. Four primers, when used simultaneously in PCR amplification, were expected to yield two amplicons in homozygous wildtype individuals and three amplicons in individuals carrying at least one copy of the mutant allele. For genotyping by PCR, a reaction volume of 25 µL was prepared with 1 x PCR buffer (1.5 mM MgCl_2_) (Thomas Scientific, Swedesboro, NJ), 0.6 mM dNTPS (0.15 mM each nucleotide), 0.4 µM left_BP_F primer and right_BP_R1/2 primer, 0.8 µM left_BP_R primer and right_BP_F primer, 1.25 U Choice Taq polymerase (Thomas Scientific) and 25–50 ng template DNA. PCR products were amplified using an Applied Biosystems Veriti thermal cycler (Foster City, CA) with the following conditions: 94 °C for 3:00 min denaturation, followed by 35 cycles at 94 °C for 1:00 min, 58 °C for 1:00 min, and 72 °C for 1:00 min, which ended with 72 °C for 10:00 min and 4 °C hold. For electrophoresis, a 1.25% (w/v) agarose gel in 1x TAE buffer was prepared with samples separated at 70 V for 90 minutes. Each PCR amplicon in a known affected cell line and a random bred negative control was gel extracted and Sanger sequenced at the MU DNA Core on an ABI 3730XL (ABI, Foster City, CA) and assembled into reference-based contigs using Sequencher (GeneCodes, Ann Arbor, MI). Predicted amplicon sequences were extracted from Felis_catus_9.0 and used as the reference for contig assembly.

## Results

### Semen cryopreservation and laparoscopic oviductal artificial insemination

Electroejaculation of the CHS-carrier male cat recovered a moderate volume (165 µl) of seminal fluid containing highly concentrated spermatozoa (138 × 10^6^ sperm/ml or 22.8 × 10^6^ total sperm). In the raw ejaculate, 70% of spermatozoa were progressively motile with a rate of progressive motility (RPM) of 3.0 (on scale of 1 to 5), and 35% of sperm exhibited normal structural morphology. The major abnormalities included bent tails and bent mid-pieces (~30% of all sperm). Following semen processing and cooling, 32 sperm pellets, each containing ~0.75 × 10^6^ motile sperm, were cryopreserved. Thawing of a single pellet at 10 months after cryopreservation revealed progressive sperm motility of 40%, indicating that each pellet should contain ~0.43 × 10^6^ motile sperm post-thaw. Frozen semen was stored for seven years prior to use with LO-AI procedures.

At the time of laparoscopy, two of the three synchronized female cats exhibited multiple fresh corpora lutea on both ovaries, without the presence of any anovulatory follicles (Table 1). In contrast, the first female evaluated laparoscopically, at 30 hours post-pLH, possessed only mature peri-ovulatory follicles and no corpora lutea. All three females were inseminated bilaterally in both oviducts with frozen-thawed semen from a single ejaculate, with each female receiving a total of 2.2–3.3 million motile sperm (Table 1). Eight semen pellets were thawed for each AI procedure, with half of the concentrated sperm sample deposited into each oviduct. Immediately after thawing, sperm progressive motility for all samples was 40%, with an RPM of 2.5, and acrosome staining of one thawed sample revealed that 32% of sperm had intact acrosomal membranes. For all females, insemination of both oviducts was completed within 20 minutes of semen thawing. Following LO-AI of the anovulatory female (11WBG35), four mature follicles on the right ovary were manually ruptured with a catheter stylet to ensure ovulation and increase the opportunity for fertilization. Ultrasonography at 23 days post-AI determined that both ovulatory females conceived as shown by the presence of gestational sacs with viable fetuses, whereas the anovulatory female showed no evidence of implantations (Table 1). The two pregnant females progressed to term without complication, with fetal development and viability monitored via ultrasonography. One queen (12ODH5), a multiparous female, went into labor at 64 days post-AI, giving birth to five healthy kittens and one stillborn kitten before experiencing dystocia due to presumed uterine inertia. The remaining kittens were delivered via C-section, with 13 kittens born (nine viable, four stillborn). The second queen (12OTN2), a primiparous female, went into labor at 66 days post-AI, but experienced dystocia during parturition of the first kitten, and two viable kittens were delivered by C-section. Viable kittens averaged (+S.D.) 96.7 + 9.0 grams body weight at birth. Fostering of three kittens from the first queen to the second to reduce lactational stress, combined with initial supplemental feeding of kittens, resulted in all 11 viable kittens surviving through weaning. All kittens were transferred from the Cincinnati Zoo & Botanical Garden to MU at four months of age.

**Table 1:** Ovarian responses and pregnancy results in domestic cats following fixed-time LO-AI with frozen-thawed semen from a Chediak Higashi Syndrome carrier male.

Female ID	11WBG35	12ODH5	12OTN2
No. ovarian follicles	14^a^	0	0
No. corpora lutea	0	52	15
No. motile sperm (x10^6^)	3.3	2.2	2.6
Pregnancy (Y/N)^b^	N	Y	Y
Gestation length (days)	—	64	66
No. full-term kittens	—	13	2
No. viable kittens^c^	—	9	2

^a^Four follicles were manually ruptured post-insemination.

^b^Ultrasonography conducted at 23 days post-AI.

^c^Five kittens were born via vaginal delivery and six kittens were delivered via C-section.

### Tandem segmental duplication in *LYST* is associated with CHS in cats

**Figure 2:**
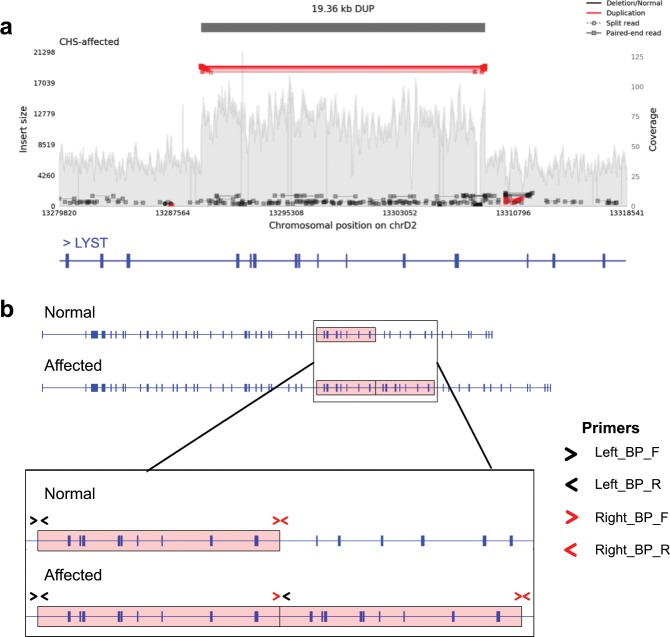
CHS in felines is associated with a 20 kb tandem segmental duplication in *LYST*. (**a**) Samplot output showing increased coverage across *LYST* exons 30 through 38 with reverse orientation read pairs colored in red spanning the duplicated region. (**b**) Schematic of NM_001290242.1:c.8347-2422_9548 + 1749dup in normal and affected individuals. Introns are depicted as a thin blue line and exons are depicted as thick blue lines. Duplicated region is highlighted in red and is ~20 kb in length. Zoomed in region shows primer pairs overlapping duplication breakpoints. Note, primers Right_BP_F and Left_BP_R produce an amplicon in affected/carrier samples that was absent in normal samples.

Whole genome sequencing of a fibroblast cell line derived from a homozygous affected cat from the original CHS colony, produced a mean coverage of 43× . *LYST* in cats is located at chrD2:13199206–13347218 and predicted to be comprised of 51 exons. Visual analysis of reads mapped to the *LYST* locus showed increased coverage across several exons that were spanned by discordant read pairs (Fig. 2a). Relative to the cat reference genome, these results were consistent with a tandem segmental duplication encompassing exons 30 through to 38 (NM_001290242.1:c.8347-2422_9548 + 1749dup) (NC_018733.3:g.13289500_13308861dup). In addition, a number of reads near the breakpoints had a read mate that mapped to a SINEC_Fc transposable elements elsewhere in the genome, suggesting the presence of a SINEC_Fc element between the duplicated copies.

To validate the mutation, primers were designed to produce three distinct amplicons, the left and right breakpoints of the duplicated region and a central breakpoint only found in samples carrying the mutant allele. The primers spanning the left breakpoint produce a 462 bp amplicon, while the primers for the right breakpoint produce either a 244 bp or 343 bp amplicon depending on the primer combination used. The primers for the central breakpoint produce a 639 bp product, which contains a SINEC_Fc element (Fig. 2b, Supplementary Table S1) (GenBank id MN240554). PCR validation produced amplicons concordant with the affected status of a positive control fibroblast cell line and normal status of a random bred negative control (Supplementary Fig. S1). Assembled Sanger sequencing for each amplification product was consistent with predicted amplicon sequence. These results demonstrated the presence of the variant allele in a separate affected cat cell line and validate the existence of breakpoints consistent with NM_001290242.1:c.8347-2422_9548 + 1749dup.

**Figure 3:**
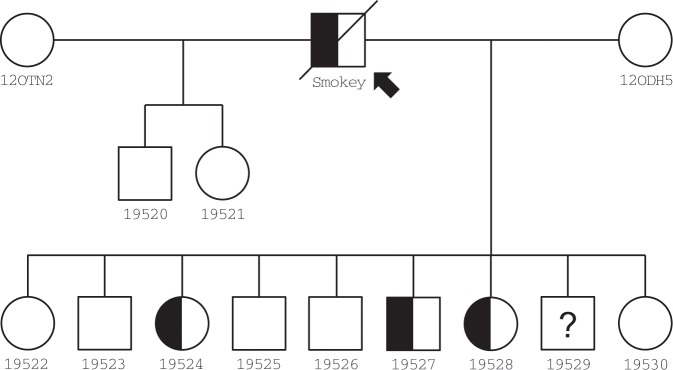
Pedigree of resurrected feline model of Chediak-Higashi syndrome via assisted reproduction. Half-filled symbols in pedigree represent predicted carrier status based on presence of the mutant allele from genotyping. The founder male Smokey (arrow) is heterozygous, as he did not present with CHS-associated symptoms throughout his life. The founder females, 12OTHN2 and 12ODH5, likely do not carry NM_001290242.1:c.8347-2422_9548 + 1749dup as these females were from a commercial breeding colony of cats. Displayed genotypes were determined using PCR screening, except for 19529, which did not produce any amplicons and whose genotype remains unknown. Lab ID (Fcat) numbers are presented under each individual in the pedigree.

DNA from additional fibroblast cell lines derived from known carriers and affected individuals from the original colony and the offspring produced from AI were genotyped for the presence of NM_001290242.1:c.8347-2422_9548 + 1749dup. The variant allele was present in all fibroblast cell lines. For the 11 viable offspring produced from AI, two females and one male, all from the same litter, contained NM_001290242.1:c.8347-2422_9548 + 1749dup as likely carriers for CHS (Fig. 3, Supplementary Fig. S2). Therefore, these results are consistent with successful resurrection of a previously extinct disease model in cats.

## Discussion

Chediak-Higashi Syndrome is a well-characterized lysosomal disease caused by mutations in *lysosomal trafficking regulator* (*LYST*). The classic diagnostic feature of the syndrome is enlarged lysosome-related organelles (LROs) in many cell types, including lysosomes, melanosomes and cytolytic granules. Other diseases associated with the regulation of LRO size and/or vesicle trafficking, such as asthma, urticaria and *Leishmania amazonensis* infections, could benefit from understanding the molecular function of *LYST* and identification of its interacting partners may provide therapeutic targets^64^. Unfortunately, the cat model for CHS was lost to the research community before the causative mutation was discovered.

CHS in the domestic cat was first observed in 1977 in a family of Persian cats, clearly demonstrating an autosomal recessive disease with similar presentation to the human condition^19,21,22^. Complementation studies suggested the human and cat conditions were likely allelic^50^. After the CHS colonies at Colorado State and Washington State University were dissolved, years later, the semen donor, Smokey, the last known living representative of this feline model for CHS, was found residing in a University of California, Davis cat colony. However, because of his advanced age and lack of libido, he was unable to naturally breed females. Electroejaculation showed that his testes continued to produce moderate quantities of viable spermatozoa with sufficient progressive motility. Although his sperm morphology was later classified as teratospermic with just 35% normal spermatozoa^65^, freezing and storage of this ejaculate ensured his genetic potential was preserved, provided the frozen-thawed spermatozoa was capable of fertilizing oocytes *in vitro* or *in vivo*. The recent development of LO-AI in cats, combined with the incorporation of oral progestin treatment for precise ovarian synchronization, provided investigators with an efficient strategy for generating viable offspring using this limited genetic resource^58,66^. As shown by the birth of multiple kittens following LO-AI, post-thaw sperm functionality was adequate to achieve the primary goal of model regeneration, requiring just two to three million motile sperm per procedure. Despite the limited amount of frozen semen, the suboptimal sperm morphology and the relatively low post-thaw sperm motility and acrosomal status, fixed-time LO-AI still proved adequate for model propagation with two of three females conceiving and the delivery of eleven viable kittens. The occurrence of dystocia was likely due to the exceptionally large litter size (13 kittens) and associated prolonged labor in one queen, a multiparous female who had previously given birth to six viable kittens via vaginal delivery during a previous LO-AI pregnancy. The second pregnant female was primiparous and possibly experienced obstructive dystocia due to a relatively narrow birth canal and slightly larger kittens (102–108 grams body weight). Dystocia reportedly occurs in three to six percent of pregnancies in the general cat population^67^. Generally, pregnancies produced by LO-AI in the Cincinnati Zoo & Botanical Garden’s cat research colony do not appear more prone to dystocia than pregnancies resulting from natural breeding^66^.

For biomedical research and drug development, the current standard is to use rodent models^68^. However, 95% of drugs fail at human trials, leading to costs of nearly $2 billion and 10 years of time per FDA drug approval^69,70^. Large animals, including cats, dogs and pigs, support drug trials, provide additional evidence for deciding to abandon drug development or proceed to human trials. This work provides a “proof-of-principle” demonstrating that cryopreservation and reproductive technologies are now well established in domestic cats, which may lead to the development of genome-edited cats as models for specific diseases. Importantly, future applications of genome-editing will likely provide the best animal model for a given disease and drug development trial, ultimately improving the efficiency and cost effectiveness of the drug development process. This model of feline CHS was selected because no other living animals were carrying the disease at the time the LO-AI procedures were performed. Hence, these ART procedures did not support animal production that could have otherwise been achieved through normal mating. Investigators interested in CHS should now realize the cat model may be available for research. In addition, study results reinforce the need for more routine cryobanking of valuable cat models, since only one CHS carrier cat had cryopreserved gametes available for model rederivation.

CHS in the feline model is likely caused by the identified 20 kb tandem segmental duplication that spans *LYST* exons 30 through 38 (NM_001290242.1:c.8347-2422_9548 + 1749dup). Discovery of the candidate causative mutation had been hampered by the availability of appropriate feline genomic resources and the complexity of the mutation itself, which required the use of WGS of an affected cat sample to efficiently resolve. A potential mechanism for the mutation is non-allelic homologous recombination, which is known to lead to either deletions or duplications and is implicated in a variety of human genomic disorders^71^. Examples include Charcot-Marie-Tooth disease type 1 A and Hereditary Neuropathy with Liability to Pressure Palsies^72,73^. Accumulation of high copy repeated DNA sequences, such as SINEC_Fc elements, around a single locus increase a region’s susceptibility to non-allelic homologous recombination.

One of the limitations of the current genetic analysis is the zygosity of the mutant allele was not determined. Since many molecular techniques require unique priming sites, determining copy number of low copy large tandem repeated sequences is challenging. Also due to the complexity of the mutation, the analyses performed were not able to determine if the haploid copy number of the duplicated region was greater than two copies. Regardless, the NM_001290242.1:c.8347-2422_9548 + 1749dup was amplified in the AI offspring, showing it was successfully inherited. Consequently, more sophisticated techniques for determining the total copy number of the duplicated region, such as RT-PCR, were not required for this analysis.

In this study, two of the primary benefits of assisted reproduction for management of cat hereditary disease models were demonstrated, specifically, 1) the propagation of individuals incapable of natural breeding due to physiological or behavioral incompatibilities, and, 2) the production of offspring following cryopreservation and long-term storage of frozen semen. Furthermore, a candidate causative mutation for the cat model of CHS, NM_001290242.1:c.8347-2422_9548 + 1749dup, was discovered in *LYST* and used to screen AI offspring to show the feline model for CHS could be successfully resurrected. The allele was successfully inherited from cryopreserved gametes using AI and produced three carrier kittens, allowing for the once extinct feline model for CHS to be fully resurrected. This study supports the use of cryopreservation for long-term maintenance of future feline biomedical models of genetic disease.

## Supplementary information

Supplementary Material.

## Data Availability

WGS data can be accessed from the Sequence Read Archive (SRA) under the BioProject accession PRJNA557464.
