# Supplementary material for: Assisted reproduction mediated resurrection of a feline model for Chediak-Higashi syndrome caused by a large duplication in LYST
Source: Sci Rep. 2020 Jan 9;10:64. doi: 10.1038/s41598-019-56896-9 (PMC6952417; doi:10.1038/s41598-019-56896-9)
Supplement: Supplementary file 1 — Supplementary Material. [file 41598_2019_56896_MOESM1_ESM.docx]

**Assisted reproduction mediated resurrection of a feline model for Chediak-Higashi syndrome caused by a large duplication in *LYST*.**

R.M. Buckley, R.A. Grahn, B. Gandolfi, J.R. Herrick, M.D. Kittleson, H.L. Bateman, J. Newsom, W.F. Swanson_,_ D.J. Prieur, and L.A. Lyons

**Supplementary Material**

**Supplementary Table S1: Primer pairs and expected amplicon sizes.**

| **Amplicon** | **Size (bp)** | **Primer** | **Sequence 5’🡪3’** |
| --- | --- | --- | --- |
| Left breakpoint | 463 | Left_BP_F | accttcttgtgccttttagagc |
|  |  | Left_BP_R | tgttttcagccctcaaaatactg |
| Right breakpoint^a^ | 245 | Right_BP_F | gtatacttggggaatgttaggattg |
|  |  | Right_BP_R | ccaatggtgaccagcaaac |
|  | 343 | Right_BP_F | gtatacttggggaatgttaggattg |
|  |  | Right_BP_R2 | gtcacatgctcctctgactaagc |
| Central breakpoint^b^ | 639 | Right_BP_F | gtatacttggggaatgttaggattg |
|  |  | Left_BP_R | tgttttcagccctcaaaatactg |

^a^Right breakpoint was amplified with two different reverse primers, producing two alternative amplicon sizes distinct from all other primer combinations. ^b^Breakpoint is only found in individuals carrying the variant allele

**Supplementary Figure S1. PCR amplification and validation of *LYST* variant breakpoints.** Individual primer pairs for each breakpoint (BP) were amplified for a wildtype negative control and an affected cell line. Lanes marked N were no template controls, lanes marked W were non-affected wildtype (Fcat 4649), and lanes marked A were affected cell line (Fcat-22502). For right breakpoint amplification, the primer pair Right_BP_F and Right_BP_R was used. For each primer pair, amplicons were consistent with expected sizes. As expected, the central breakpoint only amplified in the affected sample, indicating the presence of a tandem duplication within *LYST*. The lane marked L contains 100 bp PLUS DNA ladder (Gold Bio, St. Louis, MO) for determining amplicon size. Electrophoresis was performed on a 1.25% agarose gel at 70V for ~90 min with ethidium bromide staining.


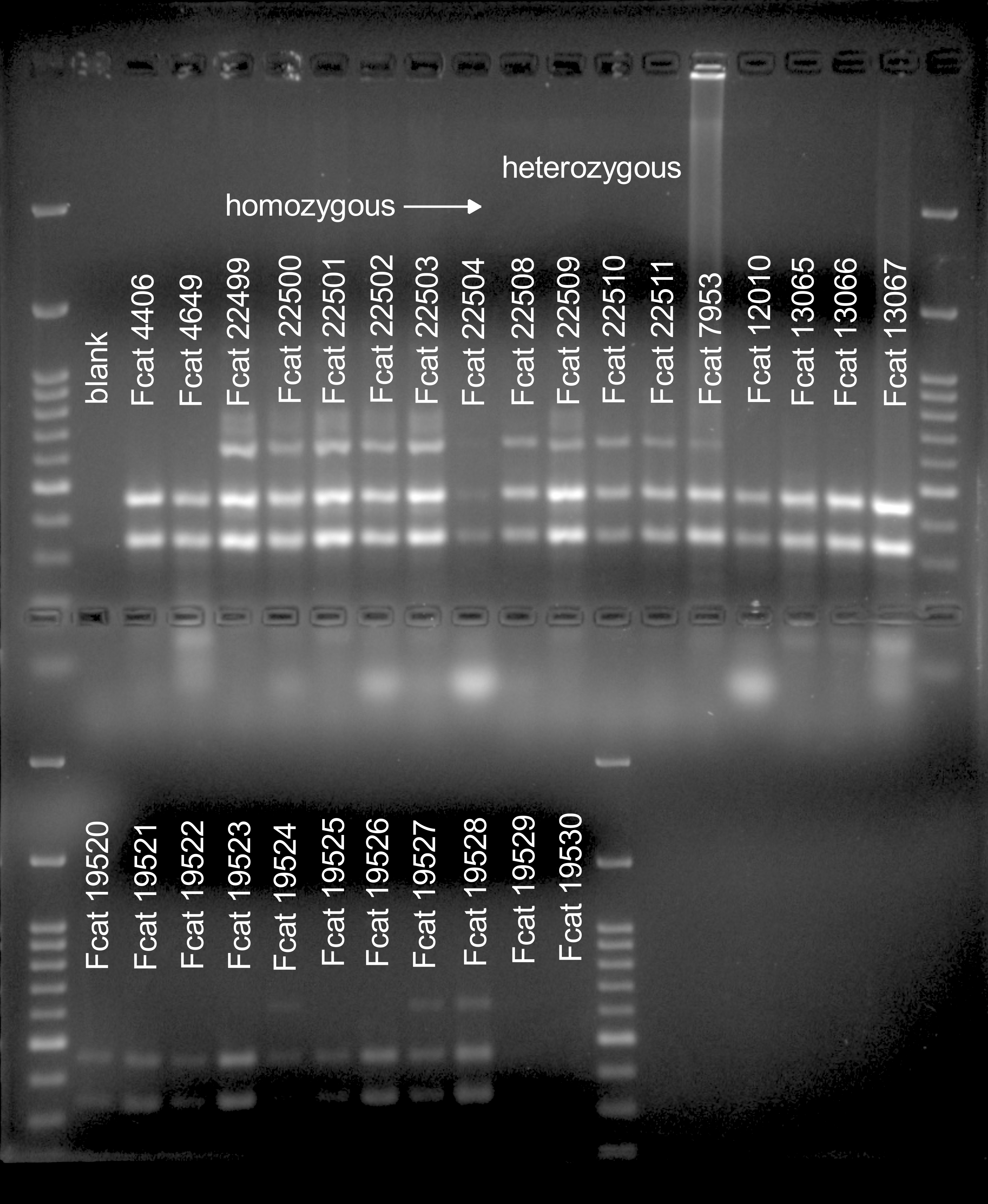


**Supplementary Figure S2. Genotyping for the feline Chediak-Higashi Syndrome variant in *LYST****.* Multiplex PCR using 4 primers simultaneously to detect breakpoints consistent with NM_001290242.1:c.8347-2422_9548+1749dup. Two amplicons at 343 bp (right breakpoint) and 462 bp (left breakpoint) represent a homozygous wildtype genotype while the presence of the third 639 bp amplicon represents the presence of the mutant allele in either a heterozygous or homozygous state. The lane titled “blank” is a no template control. Fcat 4406 and Fcat 4649 are known homozygous wildtype samples. Fcat 22499 – Fcat 22504 are known affected samples from the original CHS colony and are expected to be homozygous for the mutant allele. Fcat 22508 – Fcat 2251 are known carrier samples from the original CHS colony and are expected to be heterozygous for the mutant allele. Fcat 7953 is Smokey, the lone surviving carrier male after the original CHS colony was dissolved. Fcat 12010 – Fcat 13067 are unaffected descendants of Smokey that were produced prior to LO-AI was performed. Fcat 19520 – Fcat 19530 are offspring from LO-AI. Results show the variant allele was successfully inherited in three of nine successfully genotyped kittens. Subsequent analyses were able to provide a genotype for Fcat 19530 but not for Fcat 19529. A 100 bp PLUS DNA ladder (Gold Bio, St. Louis, MO) was used to determine amplicon size. Electrophoresis was performed on a 1.25% agarose gel at 70V for ~90 min with ethidium bromide staining.
